# High prevalence of extended-spectrum ß-lactamase producing *enterobacteriaceae* among clinical isolates in Burkina Faso

**DOI:** 10.1186/s12879-016-1655-3

**Published:** 2016-07-11

**Authors:** Abdoul-Salam Ouedraogo, Mahamadou Sanou, Aimée Kissou, Soufiane Sanou, Hermann Solaré, Firmin Kaboré, Armel Poda, Salim Aberkane, Nicolas Bouzinbi, Idrissa Sano, Boubacar Nacro, Lassana Sangaré, Christian Carrière, Dominique Decré, Rasmata Ouégraogo, Hélène Jean-Pierre, Sylvain Godreuil

**Affiliations:** Centre Hospitalier Universitaire Souro Sanou, BP 676 Bobo Dioulasso, Burkina Faso; Centre Hospitalier Universitaire Pédiatrique Charles de Gaulle, Ouagadougou, Burkina Faso; Centre Hospiatlier Universitaire Yalgado Ouédraogo, Ouagadougou, Burkina Faso; Centre Hospitalier Régional Universitaire (CHRU) de Montpellier, Département de Bactériologie-Virologie, Montpellier, France; Université Montpellier 1, Montpellier, France; INSERM U1058 “Infection by HIV and by agents with mucocutaneous tropism: from pathogenesis to prevention” and Department of Bacteriology-Virology, CHU Arnaud de Villeneuve, 371 avenue du doyen Gaston Giraud, 34295 Montpellier Cedex 5, France; CIMI, team E13 (bacteriology), Sorbonne University, UPMC Université Paris 06 CR7, F-75013 Paris, France; INSERM U1135, CIMI, team E13, Paris, France; AP-HP, Microbiology, St-Antoine Hospital, Paris, France

**Keywords:** *Enterobacteriaceae*, ESBL, Clinical samples, Inpatient and outpatient, Burkina Faso

## Abstract

**Background:**

Nothing is known about the epidemiology and resistance mechanisms of extended-spectrum ß-lactamase-producing *Enterobacteriaceae* (ESBL-PE) in Burkina Faso. The objective of this study was to determine ESBL-PE prevalence and to characterize ESBL genes in Burkina Faso.

**Methods:**

During 2 months (June-July 2014), 1602 clinical samples were sent for bacteriologic investigations to the microbiology laboratories of the tree main hospitals of Burkina Faso. Isolates were identified by mass spectrometry using a matrix-assisted laser desorption ionization-time of flight (MALDI-TOF) BioTyper. Antibiotic susceptibility was tested using the disk diffusion method on Müller-Hinton agar. The different ESBL genes in potential ESBL-producing isolates were detected by PCR and double stranded DNA sequencing. *Escherichia coli* phylogenetic groups were determined using a PCR-based method.

**Results:**

ESBL-PE frequency was 58 % (179 strains among the 308 *Enterobacteriaceae* isolates identified in the collected samples; 45 % in outpatients and 70 % in hospitalized patients). The CTX-M-1 group was dominant (94 %, CTX-M-15 enzyme), followed by the CTX-M-9 group (4 %). ESBL producers were more often found in *E. coli* (67.5 %) and *Klebsiella pneumoniae* (26 %) isolates. *E. coli* isolates (*n* = 202; 60 % of all *Enterobacteriaceae* samples) were distributed in eight phylogenetic groups (A = 49, B1 = 15, B2 = 43, C = 22, Clade I = 7, D = 37, F = 13 and 16 unknown); 22 strains belonged to the sequence type ST131. No association between a specific strain and ESBL production was detected.

**Conclusions:**

This report shows the alarming spread of ESBL genes in Burkina Faso. Public health efforts should focus on education (population and healthcare professionals), surveillance and promotion of correct and restricted antibiotic use to limit their dissemination.

## Background

The emergence and spread of Multidrug Resistant (MDR) bacteria are major public health threats. Particularly, bacteria that produce extended-spectrum ß-lactamases (ESBL) are of great concern because their resistance to penicillins and narrow- and extended-spectrum cephalosporins reduces considerably the treatment options [1]. ESBL genes originally evolved from the ß-lactamase *TEM-1*, *TEM-2* and *SHV-1* genes through mutations of the amino acids surrounding the active site and were mainly detected in nosocomial pathogens [2]. However, during the past decade, the rapid and massive spread of CTX-M-type ESBLs has been described worldwide. This has considerably changed their epidemiology because they combine the expansion of mobile genetic elements with specific clonal dissemination [3]. Furthermore, such plasmids typically carry resistance genes also to other drugs, such as aminoglycosides and fluoroquinolones [2]. Recent studies suggest that CTX-M-type ESBL- producing *Enterobacteriaceae* (ESBL-PE) are endemic in most countries of Europe, Asia and South America, with high rates of CTX-M-type ESBL-producers particularly among *Escherichia coli* (30 to 90 %) and *Klebsiella pneumoniae* (10 to 60 %) [4, 5]. Despite these public health concerns, little is known about ESBL diffusion in Africa. ESBL-PE rates between 8.8 and 13.1 % were reported in South Africa [6] and an alarmingly high proportion of ESBL-PE (49.3 %) was found among clinical isolates from Ghana [7]. Conversely, no information is available on the epidemiology of ESBL-producing pathogens in hospital or community settings in Burkina Faso, a low-income country close to Ghana. Therefore, the aim of the present study was to estimate ESBL occurrence in clinical samples from hospitalized and non-hospitalized patients and to characterize the ESBL genes as well as the genetic background of the identified *E. coli* strains.

## Methods

### Study setting

During 2 months (June–July 2014), all consecutive clinical samples sent to the microbiology laboratories of the three main hospitals of Burkina were investigated. Specifically:

1. Yalgado Ouedraogo Teaching Hospital (CHU-YO) is the largest medical institution located in Ouagadougou, the capital city with a population of about 2 million inhabitants. This hospital has 716 beds and intensive care units that are used for surgical, medical and trauma emergencies. Annually, more than 20,000 inpatients (children and adults) are admitted among 126,000 consultations.
2. Souro Sanou Teaching Hospital (CHU-SS) is the major healthcare and referral centre for the southern and western regions of Burkina Faso. It has 521 beds distributed in different specialized (medicine, surgery, gynaecology obstetric and paediatric) acute care units. The annual number of hospitalizations ranges from 15,000 to 20,000 patients among 108,000 consultations.
3. Charles de Gaulle Paediatric Teaching Hospital (CHUP-CDG) is the referral paediatric hospital in Ouagadougou with 120 beds. About 6000 children are seen each year and 5000 are hospitalized. The microbiology laboratory also receives samples from adult outpatients.

### Specimen collection, identification and antimicrobial susceptibility testing

In June and July 2014 (CHU-SS and CHUP-CDG) and July 2014 (CHU-YO), 1602 clinical samples were sent to the three microbiology laboratories for bacteriologic investigations (CHU-YO: *n* = 521, CHU-SS: *n* = 528 and CHUP-CDG: *n* = 553). Bacterial cultures could be obtained only from 584 of these samples (mainly because of poor pre-analytical sample handling) and they included 308 *Enterobacteriaceae* isolates. *Enterobacteriaceae* isolates were recovered from urine (*n* = 185), pus (*n* = 56), aspirates from various anatomic sites (*n* = 38), stool (*n* = 16), blood (*n* = 8), vaginal swabs (*n* = 3) and cerebrospinal fluid samples (*n* = 2). The remaining 276 isolates included Gram positive cocci (*Staphylococcus* spp and *Streptococcus* spp) and Gram negative bacilli (e.g., *Pseudomonas aeruginosa* and *Acinetobacter baumanii*). Species identification was performed by matrix-assisted laser desorption ionization-time of flight (MALDI-TOF) mass spectrometry (Bruker Daltonics, Bremen, Germany). Antimicrobial susceptibility was tested with the disk diffusion method on Müller-Hinton agar. The following antibiotics were tested: penicillins (amoxicillin, amoxicillin-clavulanic acid, piperacillin, piperacillin-tazobactam, ticarcillin, ticarcillin-clavulanic acid), monobactam (aztreonam), oxacephem (moxalactam), extended-spectrum cephalosporins (cefepime, cefotaxime, cefpirome, cefpodoxime, cefoxitin, ceftazidime, cephalotin), carbapenems (imipenem), aminoglycosides (amikacin, gentamicin, netilmicin and tobramycin), quinolones (nalidixic acid, ciprofloxacin, levofloxacin, ofloxacin) fosfomycin, chloramphenicol, tetracycline and trimethoprim-sulfamethoxazole. Results were interpreted according to the European Committee on Antimicrobial Susceptibility Testing (EUCAST) clinical breakpoints (Version 5.0) (http://www.eucast.org/clinical_breakpoints/). ESBL production was detected by using the combined double-disk synergy method [8]. In case of high-level cephalosporinase production, the combined double-disk synergy test was performed using cloxacillin-supplemented medium. Ertapenem minimal inhibitory concentrations (MIC), determined using the Etest (bioMerieux), were determined for all isolates.

### Molecular identification of ESBL genes

DNA was extracted from one single colony for each isolate in a final volume of 100 μL of distilled water by incubation at 95 °C for 10 min followed by a centrifugation step. The presence of *bla*_CTX-M_ (CTX-M group 1, 2, 8, 9 and 25), *bla*_TEM_, *bla*_SHV_ and *bla*_OXA-like_ genes was assessed by multiplex PCR according to a previously published method [9]. DNA from reference *bla*_CTX-M_, *bla*_TEM_, *bla*_SHV_ and *bla*_OXA-like_-positive strains was used as positive control. PCR products were visualized after electrophoresis on 1.5 % agarose gels containing ethidium bromide at 100 V for 80 min. A 100 bp DNA ladder (Promega, USA) was used as a marker size. PCR products were purified using the ExoSAP-IT purification kit (GE Healthcare, Piscataway, NJ, USA) and sequenced bidirectionally on a 3100 ABI Prism Genetic Analyzer (Applied Biosystems). Nucleotide sequence alignment and analyses were performed online using the BLAST program available at the National Center for Biotechnology Information web page http:// www.ncbi.nlm.nih.gov.

### PCR detection of *Escherichia coli* phylogroups and ST131

*E. coli* phylogenetic grouping was performed using the PCR-based method described by Clermont and al. [10]. For strains assigned to the B2 phylogenetic group, the sequence type (ST) 131 was determined using a PCR method specific for the O25-b serotype with primers that target the *pab*B and *trp*A genes, as previously described [11].

### Statistical analysis

Statistical analysis was performed with Epi Info, version 3.5.3 [Centers for Disease Control and Prevention (CDC), Atlanta, GA, USA]. Associations between demographic variables (sex, site of infection and age) and infection by ESBL-PEs were analysed by using odds ratio and a multinomial logistic regression model, when appropriate. A value of *p* < 0.05 was considered to be statistically significant.

## Results

### Occurrence of ESBL-producing *enterobacteriaceae*

During the study period, 308 different enterobacterial isolates were recovered from 158 hospitalized and 150 non-hospitalized patients (Table 1). The mean age of these patients was 29.7 ± 24.6 years and the sex ratio 1.4; 118 patients (38 %) were younger than 15. Among these 308 isolates, 179 (58 %) were identified as potential ESBL-PEs by antimicrobial susceptibility testing. PCR analysis confirmed that they all carried ESBL genes (Table 1). Considering the isolate origin, ESBL-PE prevalence was of 65 % (42/65) at CHU-YO, 59 % (84/142) at CHU-SS and 52 % (53/101) at CHUP-CDG. Moreover, ESBL-PEs were found in 45 % of outpatients and 70 % of hospitalized patients (*p* < 0.001). In hospitalized patients, no demographic factor was significantly associated with ESBL-PE occurrence (*p* > 0.05) (Table 1). Conversely in outpatients, the ESBL-PE prevalence was significantly higher among patients older than 65 years of age (Odd Ratio [OR] = 6.4, 95 % CI = 0.47–86.34; *p* < 0.001). ESBL-PE rate was also significantly higher in male than female outpatients (OR = 4.59) and in urinary samples (59 of 119; 50 %) (Table 1). Species identification showed that the 179 ESBL-PEs included 121 (67.5 %) *E. coli*, 46 (26 %) *K. pneumoniae,* 7 (4 %) *Enterobacter cloacae,* 2 (1 %) *Providencia stuartii*, 1 (0.5 %) *Enterobacter aerogenes*, 1 (0.5 %) *Citrobacter freundi* and 1 (0.5 %) *Morganella morgannii* species (Table 2). The highest proportion of ESBL-PEs was found in blood samples (6/8, 75 %). Moreover, within each species, the fraction of ESBL producers was highest among *Morganella morgannii* isolates (100 %), followed by *K. pneumoniae* (66 %) and *E. coli* (60 %) (Table 2). The 129 non-ESBL-PEs included *E. coli* (81/2002, 40 %), *K. pneumoniae* (24/70, 34 %) *Enterobacter cloacae* (6/13, 46 %), *Providencia stuartii* (4/6, 66 %) *Enterobacter aerogenes* (2/3, 77 %) *Citrobacter freundi* (2/3, 66 %), *Salmonella spp* (3/3, 100 %), *Proteus mirabilis* (5/5, 100 %) and *Leclercia adecarboxylata* (1/1) species.

**Table 1:** Demographic characteristics and source of the bacterial isolates

	Outpatients (*n* = 150)				Inpatients (*n* = 158)			
Variable	ESBL-positive	ESBL-negative	Odds Ratio (95 % CI)	*P*-value	ESBL-positive	ESBL-negative	Odds Ratio (95 % CI)	*P*-value
	(*n* = 68)	(*n* = 82)			(*n* = 111)	(*n* = 47)		
Sex				<0.001				0.724
F (*n* = 129)	16	48	1		47	18	1	
M (*n* = 179)	52	34	4.59 (2.14–9.84)		64	29	0.85 (0.42–1.70)	
Source of isolates				0.019				0.139
Urine sample (*n*–185)	59	60	1		48	18	1	
Pus (*n* = 56)	06	07	0.87 (0.28–2.75)		32	11	1.09 (0.46–2.61)	
Aspirate (*n* = 38)	02	03	0.68 (0.11–4.20)		24	09	1.00 (0.39–2.56)	
Other^a^ (*n* = 29)	01	12	0.08 (0.01–0.67)		07	09	0.29 (0.09–0.90)	
Age				<0.001				0.607
≤28 days	01	02	1		09	04	1	
>28 days–1 year	01	08	0.20 (0.01–4.72)		07	04	0.78 (0.14–4.27)	
>1–5 years	02	15	0.31 (0.02–5.19)		06	06	0.44 (0.09–2.28)	
>5–15 years	04	07	1.14 (0.08–16.95)		30	12	1.11 (0.29–4.31)	
>15–65 years	44	45	1.96 (0.17–22.35)		49	19	1.15 (0.32–4.17)	
>65 years	16	05	6.40 (0.47–86.34)		10	02	2.22 (0.33–15.18)	

*Abbreviations*: *CI* confidence interval, *ESBL* extended-spectrum beta-lactamase, *F* females, *M* males, *n* number

^a^Other: stool, cerebrospinal fluid, blood samples and high vaginal swabs

**Table 2:** Prevalence of ESBL-producing isolates among the different Enterobacteriaceae species identified in our samples

	Distribution of ESBL-producing isolates in samples (%)								
Species	Within species	Total	Urine	Stool	Blood	Pus	Aspirates	HVS	CSF
		(%)	(*n* = 185)	(*n* = 16)	(*n* = 18)	(*n* = 56	(*n* = 38)	(*n* = 3)	(*n* = 2)
*Escherichia coli*	121/202 (60 %)	67.5 %	67/114	0/15	0	29/39	23/31	2/2	0/1
*Klebsiella pneumonia*	46/70 (66 %)	26 %	32/51	0	5/6	6/8	3/4	0/1	0
*Enterobacter cloacae*	7/13 (54 %)	4 %	6/10	0	0	1/3	0	0	0
*Enterobacter aerogenes*	1/3 (33 %)	0.5 %	1/2	0	0	0	0/1	0	0
*Citrobacter koseri*	0/1	–	0	0	0	0	0/1	0	0
*Citrobacter freundi*	1/3 (33 %)	0.5 %	1 /2	0	0	0/1	0	0	0
*Proteus mirabilis*	0/5	–	0/4	0	0	0/1	0	0	0
*Providencia stuartii*	2/6 (33 %)	1 %	0/1	0	1/1	1/3	0/1	0	0
*Salmonella spp*	0/3	–	0	0/1	0/1	0	0	0	0/1
*Morganella morgannii*	1/1 (100 %)	0.5 %	0	0	0	1/1	0	0	0
*Leclercia adecarboxylata*	0/1	–	0/1	0	0	0	0	0	0
Total (%)	179/308 (58 %)	100 %	107/185	0/16	6/8	38/56	26/38	2/3	0/2

*Abbreviations*: *CSF* cerebrospinal fluid, *ESBL* extended-spectrum beta-lactamase, *HVS* high vaginal swab, *n* = number

### Antibiotic susceptibility patterns

**Fig. 1:**
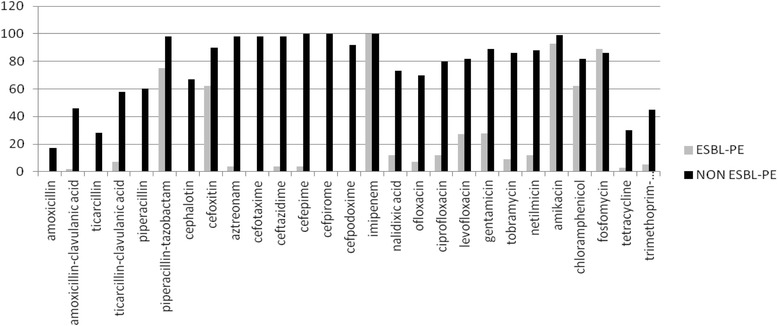
Results of the antibiotic susceptibility test for the 179 ESBL-producing (ESBL-PE) and the 129 non-ESBL-producing (NON ESBL-PE) *Enterobacteriaceae* isolates. The histogram shows the percentage of ESBL-PE and NON ESBL-PE isolates that were susceptible to each tested antibiotic compound. Y axis: Antibiotic susceptibility (%)

The susceptibility pattern of ESBL producing (*n* = 179) and non-producing (*n* = 129) *Enterobacteriaceae* isolates is shown in Fig. 1. ESBL-PE isolates were more resistant to the other tested antibiotics than non-producers: cotrimoxazole (45 % vs 5 %), gentamicin (89 % vs 27.5 %), tobramycin (86 % vs 9 %), netilmicin (88 % vs 12 %), ciprofloxacin (80 % vs 12 %), ofloxacin (70 % vs 7 %) and levofloxacin (82 % vs 27 %) (*p* < 0.05). None of the collected *Enterobacteriaceae* isolates was resistant to imipenem. Four isolates had high ertapenem MCI. Additional investigations showed that these four isolates carried blaOXA181 (47).

### Molecular characterization of ESBL and other ß-lactamase genes

Most ESBL-PE isolates (94 %) were identified as CTX-M group 1 producers because all of them carried the *bla*_CTX-M-15_ gene. CTX-M group 9 producers represented only 4 % of all ESBL-PEs (*bla*_CTX-M-14_ was detected in three isolates and *bla*_CTX-M-27_ in five samples (Table 3). The *bla*_SHV-12_ gene was detected in two isolates. The ESBL genes were detected alone or in association with one to three other ß-lactamase genes: *bla*_OXA-1_, *bla*_SHV-1_ and *bla*_TEM-1_. While *bla*_CTX-M-15_ was found in all the different enterobacterial species, *bla*_CTX-M-14_ was detected only in *E. coli* samples (*n* = 3), *bla*_CTX-M-27_ in *E. coli* (*n* = 2)*, K. pneumoniae* (*n* = 1) and *E. cloacae* (*n* = 1) isolates, and *bla*_SHV-12_ in one *E. coli* and one *K. pneumoniae* sample (Table 3).

**Table 3:** Characterization of genes encoding beta lactamases in the 179 ESBL-producer isolates

	Outpatients (*n* = 68)			Inpatients (*n* = 111)	
Isolates (*n*)	Hospital (*n*)	ESBL Type	Other ß-lactamases	ESBL Types	Other ß-lactamases
*Escherichia coli (121*)	CHU-YO (37)	CTX-M-15 (1)	OXA-1(1)	CTX-M-14 (1)	SHV-1, TEM −1 (1)
		CTX-M-15 (2)	SHV-1 (2)	CTX-M-14 (1)	TEM-1 (1)
		CTX-M-15 (1)	TEM-1 (1)	CTX-M-15 (3)	TEM-1, OXA-1 (3)
		CTX-M-15 (1)	–	CTX-M-15 (7)	TEM-1(7)
		–	–	CTX-M-15 (3)	SHV-1 (3)
		–	–	CTX-M-15 (12)	OXA-1 (12)
		–	–	CTX-M-15 (3)	–
		–	–	CTX-M-27 (2)	TEM-1 (2)
	CHUSS (52)	CTX-M-15 (3)	SHV-1, OXA-1 (3)	CTX-M-15 (1)	SHV-1, OXA-1 (1)
		CTX-M-15 (1)	TEM-1, OXA-1 (1)	CTX-M-15 (1)	TEM-1, OXA-1 (1)
		CTX-M-15 (2)	TEM-1 (2)	CTX-M-15 (1)	TEM-1 (1)
		CTX-M-15 (27)	OXA-1 (27)	CTX-M-15 (15)	OXA-1 (15)
		SHV-12 (1)	–	–	–
	CHUP-CDG (32)	CTX-M-15 (1)	OXA-1 (1)	CTX-M-14 (2)	TEM-1, OXA-1 (2)
		CTX-M-15 (4)	TEM-1(4)	CTX-M-15 (8)	TEM-1, OXA-1 (8)
		CTX-M-27 (1)	OXA-1, TEM-1(1)	CTX-M-15 (10)	TEM-1(10)
		–	–	CTX-M-15 (6)	OXA-1 (6)
*Klebsiella pneumonia* (46)	CHU-YO (5)	CTX-M-15(1)	TEM-1, OXA-1(1)	CTX-M-15 (1)	SHV-1, OXA-1, TEM-1 (1)
		SHV-12 (1)	–	CTX-M-15 (2)	OXA-1 (2)
	CHUSS (24)	CTX-M-15(1)	SHV-1, OXA-1, TEM-1(1)	CTX-M-15(3)	SHV-1, OXA-1, TEM-1(3)
		CTX-M-15(2)	SHV-1, OXA-1, (2)	CTX-M-15(2)	SHV-1, OXA-1 (2)
		CTX-M-15(3)	SHV-1, TEM-1 (3)	CTX-M-15(2)	OXA-1, TEM-1 (2)
		CTX-M-15(5)	OXA-1 (5)	CTX-M15 (2)	OXA-1 (2)
		CTX-M-15(1)	TEM-1(1)	CTX-M–15(1)	–
		CTX-M-15(2)	SHV-1 (2)	–	–
	CHUP-CDG (17)	CTX-M-15 (2)	OXA-1 (2)	CTX-M-15 (3)	OXA-1(3)
		–	–	CTX-M-15 (5)	EM-1, OXA-1 (5)
		–	–	CTX-M-15(4)	SHV-1, TEM-1(4)
		–	–	CTX-M-15(2)	SHV-11
		–	–	CTX-M-27(1)	SHV-1(1)
Other strains (12)		CTX-M-15(2)	OXA-1, TEM-1 (2)	CTX-M-15 (5)	OXA-1, TEM-1
		CTX-M-15 (2)	TEM-1, SHV-1 (2)	CTX-M-15 (1)	SHV-11(1)
		CTX-M-27 (1)	OXA-1 (1)	CTXM-15 (1)	–

*Abbreviations*: *N* number

### *Escherichia coli* phylogenetic groups and sequence type 131

The phylogenetic group analysis revealed diversity in both ESBL-producing and non-producing *E. coli* isolates (n = 202). Specifically, *E. coli* isolates belonged to eight different phylogenetic groups (A = 49, B1 = 15, B2 = 43, C = 22, Clade I = 7, D = 37 F = 13) and 16 could not be classified according to Clermont *and al.* method [10]. These 16 isolates might represent a new phylogenetic group. Phylogenetic group A was more represented among ESBL-producers (31 of 121; 26 %), followed by group D and B2 (for both: 26 of 121; 21.5 %). Non-ESBL producers belonged mainly to the phylogenetic groups A and B2 (18 and 17 of 81, respectively; 21 %). Moreover, the ST131 sequence type was detected in 16 ESBL-producers and in six non-producers (Table 4).

**Table 4:** Phylogenetic group assignment of the 202 *E. coli* strains subdivided based on the detection or not of ESBL genes

ESBL-Positive (*n* = 121)										ESBL-Negative (*n* = 81)								
	A	B1	B2	C	Clade I	D	F	Unknown	ST131	A	B1	B2	C	Clade I	D	F	Unknown	ST131
Hospital (number of samples)																		
CHU-YO (55)																		
Outpatients (16)	0	0	3	0	0	2	0	0	1	0	0	1	6	0	0	0	4	0
Inpatients (39)	9	2	4	6	0	5	6	0	3	0	0	3	0	0	2	0	2	1
CHU-SS (90)																		
Outpatients (59)	6	1	11	4	2	8	1	0	8	7	2	7	4	1	3	2	0	4
Inpatients (31)	7	1	5	1	0	3	1	1	4	2	2	1	1	1	0	1	3	0
CHUP-CDG (57)																		
Outpatients (20)	0	1	0	0	0	2	2	1	0	7	2	3	0	0	1	0	1	1
Inpatients (37)	9	2	3	0	3	6	0	3	0	2	2	2	0	0	5	0	1	0

## Discussion

In this study, we investigated the frequency of ESBL production by *Enterobacteriaceae* isolates from clinical samples sent to the three main hospitals of Burkina Faso in June and July 2014. Overall, 58 % of these isolates were ESBL-PEs. This is much higher than the rates reported in Europe [12, 13] and in other African countries: Algeria (17.7–31.4 %), Egypt (42.9 %) [14] and Ghana (49.4 %) [7]. Lack of antibiotic surveillance may have contributed to increasing the ESBL-PE problem that certainly has been present in Burkina Faso for a long time. Indeed, it has been shown that in countries with limited resources where hygiene is poor and antibiotics are misused, the absence of anti-microbial surveillance programmes increases the risk of multi-resistance development by bacteria in hospitals and in the community [15–17]. We found that blood cultures had the highest proportion of ESBL-PE isolates. This differs from the results of a recent literature review on ESBL-PE prevalence in Africa [18] showing a significantly lower proportion of ESBL-PE in blood cultures than in other specimens. This discrepancy is certainly explained by the small number of enterobacterial strains (eight of which six were ESBL-PEs) recovered from blood samples. Indeed, 107 ESBL-PEs were identified in urine samples (107/185, 58 %), a prevalence similar to what reported in previous studies [7, 19–21]. ESBL producers were more often found in *E. coli* (67 %) and *K. pneumoniae* (26 %) isolates, in agreement with previous works showing that these two species are the predominant ESBL-producers worldwide [2, 22]. ESBL-producing *E. coli* is considered to be responsible for hospital- and community-acquired infections, while ESBL-producing *K. pneumoniae* is considered mainly a nosocomial pathogen [2, 22]. In agreement, we identified ESBL-producing *K. pneumoniae* most frequently in samples from hospitalized patients. ESBL-PE prevalence differed considerably between outpatients and inpatients (45 % vs. 70 %: *p* < 0.001). More than two thirds of enterobacterial infections in hospitalized patients were thus caused by an ESBL-PE. In Burkina Faso, patients are usually hospitalized only in the case of very severe symptoms and after a long and empiric antibiotic therapy. These factors could explain this alarmingly high resistance level in hospitalized patients and also in outpatients (45 % compared with 7.5 % of community-acquired infections in Morocco [23] and 11.7 % in Nigeria) [24]. In outpatients, ESBL-PE frequency was significantly higher in isolates from older patients (more than 65 years of age, [OR] = 6.4, 95 % CI = 0.47–86.34). These results are in agreement with the study by Colodner and al. [3] showing that elderly patients present a higher antibiotic pressure and more underlying diseases, two significant risk factors for infection by ESBL producers [25]. In addition, ESBL-PE rate was significantly higher in male outpatients (OR = 4.59, 95 % CI = 2.14–9.84) and the urinary tract was the most frequent source (59 of 119, 50 %). The possible explanation may be that complicated urinary tract infections are more frequent in elderly men than elderly women [26].

In this study most ESBL-PEs were resistant to multiple drugs, especially to fluoroquinolones, aminoglycosides, cotrimoxazole and tetracycline, as described in previous studies [27–29]. This level of multi-resistance could lead to potential therapeutic impasses. Indeed, more than three quarters of ESBL-PE isolates were resistant to fluoroquinolones and aminoglycosides (but for amikacin), thus compromising the choice of antibiotic treatment, especially for outpatients with urinary tract infections. Moreover alternative antimicrobial agents, such as amikacin, fosfomycin and imipenem, are very expensive and difficult to obtain in Burkina Faso. These alarming results should act as an impetus for the establishment of antibiotic control policies. Indeed, currently, there is no restriction in the use of antibiotics in Burkina Faso. Antibiotics can be purchased over the counter without medical prescription. Patients may buy only a few tablets of an antibiotic because of limited money availability. Moreover, patients may begin an antimicrobial regimen and stop it when they feel better, before the end of the treatment, to save the remaining tablets for another time.

Finally, we found that 94 % of ESBL-PEs carried the *bla*_CTX-M-15_ gene. In the last decade, CTX-M enzymes, particularly CTX-M-15, have emerged worldwide and are the most prevalent in Europe, America and Asia [30–36]. Moreover, eight strains were CTX-M group 9 producers (*bla*_CTX-M-27_ in five and *bla*_CTX-M-14_ in three). These genes have been previously detected in *E. coli* isolates in Kenya [37] and in Egypt [38]. Nevertheless, the *bla*_CTX-M-15_ gene remains dominant in the African continent: 59 % of ESBL-PE in South Africa [34], 83 % in Mali [39], 91 % in Tunisia [40] and 96 % in Cameroon [41]. The *bla*_SHV-12_ gene (detected in one *E. coli* and one *K. pneumoniae* sample) has emerged in recent years and has been also detected in different *Enterobacteriaceae* isolates in the previously quoted studies in African countries [34, 38–41].

The phylogenetic group assignment of the 202 *E. coli* isolated showed a high diversity in both populations (out-patients and in-patients) without any association between a specific strain and ESBL production. This indicates that the high frequency of ESBL carriage is not caused by the epidemic spread of a single resistant clone. This contrasts with previous studies in which the dissemination of CTX-M-15-producing isolates was associated with the spread of the ST131 *E. coli* strain belonging to phylogenetic group B2 [42–44]. Indeed, in the present study, most isolates were assigned to the commensal groups A (49/202, 24 %) and B2 (43/202, 21 %). Only 13 % (16/121) of ESBL-producers and 7 % (6/81) of non ESBL-producers belonged to the ST131 clone. Moreover, some ESBL-producing *E. coli* isolated from urine, pus and blood samples belonged to three phylogenetic groups associated with CTX-M-15 dissemination: the virulent extra-intestinal group D (26/121) [45] and groups C (11/121) and F (10/121), usually detected in urinary tract infections [46]. This important genetic diversity among isolates suggests that the high rate of ESBL production and associated resistance are more likely caused by the diffusion of plasmids carrying antibiotic resistance genes than to cross-transmission between patients. The maintenance of these plasmids was probably favoured by antibiotic pressure. Further investigations, including multilocus sequence typing and plasmid characterization, are needed to complete this study.

## Conclusions

In summary, this first survey shows an alarmingly high frequency of multi-resistant ESBL-PEs among clinical isolates in Burkina Faso. The analysis of the resistance genes highlighted an important dissemination of *bla*_CTX-M-15_ without clonal dissemination, suggesting a strong antibiotic selection pressure in hospital and community settings. Public health efforts should focus on educating the population and healthcare professionals about the proper use of antibiotics to halt/limit the spread of multi-resistant bacteria.

## Abbreviations

CHUP-CDG, Charles de Gaulle Paediatric Teaching Hospital; CHU-SS, Souro Sanou Teaching Hospital; CHU-YO, Yalgado Ouedraogo Teaching Hospital; ESBL, extended-spectrum ß-lactamases; ESBL-PE, extended-spectrum ß-lactamase-producing *Enterobacteriaceae*; MALDI-TOF, matrix-assisted laser desorption ionization-time of flight; MDR, multidrug resistant; OR, odd ratio
